# Milliwatt-scale 3D thermoelectric generators *via* additive screen printing†

**DOI:** 10.1039/d5ee01151e

**Published:** 2025-07-04

**Authors:** Sairam Antharam, Muhammad Irfan Khan, Leonard Franke, Zirui Wang, Nan Luo, Jan Feßler, Wenjie Xie, Uli Lemmer, Md Mofasser Mallick

**Affiliations:** a Institut für Materialwissenschaft, Technical University of Darmstadt 64287 Darmstadt Germany; b Light Technology Institute, Karlsruhe Institute of Technology (KIT) 76131 Karlsruhe Germany uli.lemmer@kit.edu mofasser.mallick@kit.edu; c Institute of Microstructure Technology, Karlsruhe Institute of Technology (KIT) 76344 Eggenstein-Leopoldshafen Germany

## Abstract

Electronic components driving digitalization, such as wearables, Internet of Things (IoT), and Industry 4.0 systems, consume a growing portion of the global primary energy, largely relying on lithium-ion batteries. To enable a sustainable alternative, we explore cost-effective, fully printed thermoelectric generators (TEGs), which can be an alternative to batteries in low-power electronics. We here report a promising additive screen-printing method to fabricate two printed 3D TEGs (print-TEG I and print-TEG II) with varying thermocouple counts and a 0.36 fill factor, overcoming high contact resistance and thickness limitations. The print-TEGs were prepared *via* layer-by-layer printing of electrodes, interlayers, and n- and p-type legs, with six different layouts. Printed Ag_2_Se as n-type legs and Bi_0.5_Sb_1.5_Te_3_ as p-type legs were used for TEG fabrication. The print-TEG II with 50 thermocouples generates a maximum power output *P*_max_ of 1.22 mW with an open circuit voltage, *V*_OC_ of 268 mV for Δ*T* = 43 K. The print-TEG shows a highest power density *P*_d_ of 67 μW cm^−2^ (>400 μW g^−1^) for a fully printed planar TEG. The results demonstrate the potential of print-TEGs as a steadfast power source, guaranteeing nonstop operation of low-power electronic devices.

## Abstract 2

This study reports an additive screen-printing method for a high-performance fully printed planar 3D TEG, achieving a milliwatt-scale power output of 1.22 mW at Δ*T* of 43 K, underlining its potential for battery-free IoT applications.

## Graphical abstract

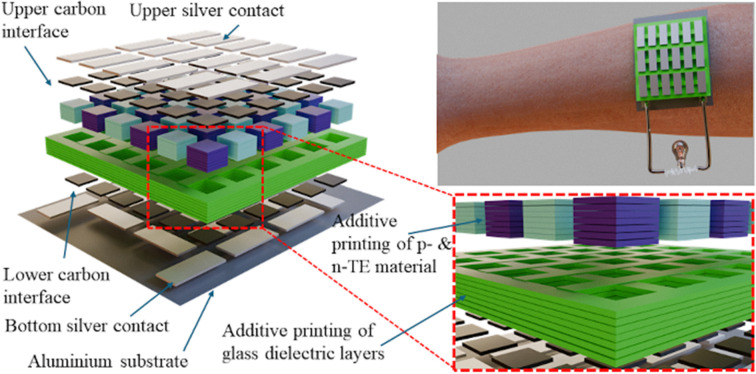

**Broader context**

Electronic systems, the backbone of digitalization such as wearables and the Internet of Things, are mainly powered by lithium-ion batteries. This work explores an alternative sustainable energy source based on a printed thermoelectric generator (TEG). Conventional TEGs, while capable of producing power of the order of watts, have limited customization and scalability-challenges that printing technology can address. However, developing high-performance fully printed TEGs is not straightforward, requiring extensive optimization of properties and dimensions of the printed legs. We here report an additive screen-printing method to fabricate fully printed 3D TEGs using Ag_2_Se (n-type) and Bi_0.5_Sb_1.5_Te_3_ (p-type), overcoming two major challenges; (a) high contact resistance and (b) thickness limitations. The TEGs are prepared *via* layer-by-layer printing of electrodes, interlayers, and TE legs. The multiple layers of p- and n-type legs were printed *via* an additive screen-printing process to attain the desired thickness, and carbon-based interfaces were printed between the TE legs and electrodes to reduce the contact resistance. As a result, a fully printed 3D TEG with 50 thermocouples yields milliwatt-scale power of 1.22 mW and a power density of 67 μW cm^−2^ for Δ*T* = 43 K. These TEGs could facilitate a steadfast power source for remote areas and lower electronic waste.

## 1. Introduction

The demand for sustainable energy supply is rising due to industrialization and the growing use of electronic equipment. Low-power wearables, IoTs, and industry 4.0 systems are also in need of a decentralized energy supply, largely powered by batteries.^1–3^ The production of these batteries not only depends on fossil fuels, but also their disposal contributes to resource depletion and pollution.^4,5^ Alternative energy sources, particularly those capable of delivering milliwatt-scale power, can offer a more sustainable solution than batteries for meeting this growing demand. Currently, over 60% of the primary energy used is wasted as heat, and more than 50% of the total waste heat is low-grade thermal energy.^6^ The thermoelectric generator (TEG) is a promising technology without moving components. It can be employed to convert the abundant heat to electric power *via* the Seebeck effect. A TEG is a solid-state device that consists of several n-type and p-type semiconductor legs connected electrically in series and thermally in parallel. When subject to a thermal gradient, a TEG produces a voltage due to charge transfer between the n-type and p-type legs.^7^ Hence, the cost-effective application of thermoelectric (TE) technology in wearables and industries, which focus on low-power solutions for automation, sensing, IoT, and industry 4.0 systems, could be transformative.^8–11^ However, commercially available TEGs are based on bulk materials, with limited shape-conformability and complex manufacturing processes. These bulk-processed TEGs are fabricated by homogenizing highly pure elements in an inert atmosphere, followed by grinding, mechanical alloying, and sintering.^12^ These processes, along with other manufacturing techniques requiring intensive labor and generating material waste, make the production of bulk TEGs costlier compared to other energy sources, including photovoltaics and batteries. Printing technologies, especially screen printing, offer a promising solution to the challenges of shape conformability and the high costs associated with bulk TEGs.^13,14^ The screen printing method facilitates the precise distribution of TE materials in customizable geometries. It also enables scalable manufacturing processes, highlighting the potential of screen-printed TEGs to prevail over conventional bulk TEGs in terms of cost-effectiveness and geometric versatility.^15^ Printed planar and folded TEGs have already shown significant potential for powering wearable devices, environmental monitoring sensors, and other low-power IoT devices.^11,15–17^ Their adaptability to different mechanical designs, such as planar, origami-inspired, and transversal configurations, makes them suitable for diverse industrial and consumer applications. Recently, Bi-Te-based screen-printed p- and n-type TE films have been used to fabricate a fully printed folded TEG that generated a normalized power density of 108 nW cm^−2^ K^−2^.^18^ Brunetti *et al.* demonstrated a screen-printed organic TEG in origami architecture, resulting in a normalized power density of 0.718 nW cm^−2^ K^−2^.^19^ A screen-printed p–n junction TEG outperforms conventional π-type printed-TEGs, with a normalized power density of 8.4 nW cm^−2^ K^−2^,^20^ while Cui *et al.* reported a normalized power density of 0.031 nW cm^−2^ K^−2^ for a printed TEG by dispenser printing.^21^ Hwang *et al.* exploited direct ink writing to fabricate an integrated 3D-compliant TEG, presenting a normalized power density of 0.124 nW cm^−2^ K^−2^.^22^

Zhang *et al.* developed a novel stamp-like paper-based TEG, where a UV-curing ink was used to fill the space in between to avoid oxidation during the process. Thus, the normalized power density was improved to a high level of 27.8 nW cm^−2^ K^−2^.^23^ Kim *et al.* first produced a flexible TEG by combining screen printing and a laser multi-scanning lift-off process.^24^ Among all of the abovementioned fabrication methods, screen printing of planar TEGs can be a cost-effective technology that facilitates the scalability of TEG fabrication, especially for large-area applications. However, high electrical contact resistance, high processing temperature, and the limited thickness achievable with screen printing restrict the power output of a fully printed TEG to the microwatt range for Δ*T* < 50 K.^12,25^

**Fig. 1:**
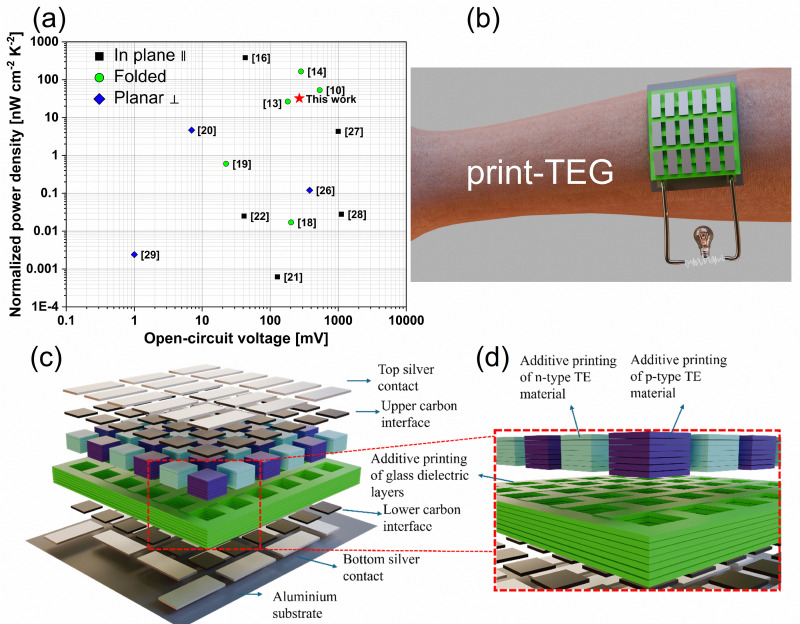
Design and performance of print-TEGs. (a) Comparison of the normalized power density of our device with recently reported fully printed TEGs, (b) schematic representation of a demonstration for wearables, (c) layer-by-layer representation of a 3D print-TEG, and (d) magnified representation of several printed layers in a 3D print-TEG.

We here report a promising additive screen-printing method together with low-temperature processing and interface engineering to fabricate two 3D TEGs with a different number of thermocouples (print-TEG I and print-TEG II), overcoming high contact resistance and thickness limitations. As a result, our fully printed 3D print-TEGs yield milliwatt-scale power at a low thermal gradient. The print-TEGs are prepared *via* layer-by-layer printing of bottom electrodes, carbon interlayers, TE legs, carbon interlayers, and top electrodes with different layouts. Here, printed Ag_2_Se n-type legs and Bi_0.5_Sb_1.5_Te_3_ p-type legs have been used for TEG fabrication. To attain the desired thickness, the p- and n-type legs were printed multiple times *via* an additive screen-printing process (*cf.*Fig. 1(c) and (d)). The 3D print-TEG II with 50 thermocouples generates a power of ∼1.22 mW and a power density of ∼67 μW cm^−2^ for Δ*T* = 43 K, rendering this device suitable for powering sensor systems. Compared with the existing fully printed TEGs,^22,26–29^Fig. 1(a) shows the highest-ever normalized power densities of 105 nW cm^−2^ K^−2^ considering only the TE leg cross section. A value of 36 nW cm^−2^ K^−2^ at Δ*T* = 43 K is calculated when the total TEG cross section is considered.

## 2. Experimental methods

### 2.1. Materials

Ingots of p-type Bi_0.5_Sb_1.5_Te_3_ (EVERREDtronics), selenium powder (200 mesh, ≥99.5% Thermo Fisher Scientific), silver powder (Sigma-Aldrich), Se powder (100 mesh, ≥99.5% trace metals basis, Sigma-Aldrich), copper powder (spheroidal) (10–25 μm, 98%, Sigma-Aldrich), polyvinylpyrrolidone (PVP) (average *M*_w_ ∼ 40 000, Sigma Aldrich), *N*,*N*-diethylformamide (DEF) (Sigma-Aldrich), terpineol (Sigma Aldrich), silver ink (Thermo Fischer), carbon ink (Dycotec), silica glass powder (Aksharchem India), and anodized aluminum substrate.

### 2.2. Preparation of printable n- and p-type TE inks

The preparation method of the TE inks follows the previously published work.^14,25^ The n- and p-type printable inks were prepared by wet milling of inorganic TE particles and an organic solution. The n-type TE particle was prepared using elemental Ag and Se powders in the stoichiometric ratio. For the p-type TE particle, Bi_0.5_Sb_1.5_Te_3_ (BST) was mixed with 5 wt% of an inorganic binder composed of elemental Cu and Se in a 2 : 1 ratio. The organic solution was prepared by mixing an organic binder, PVP, in 8 wt% with a solvent, a blend of terpineol and DEF (4 : 1). The mixture was stirred at 1000 rpm for about 10 minutes to dissolve the PVP and achieve a homogeneous solution. Finally, the two TE inks were prepared by wet milling stoichiometric n-type and p-type TE particles with the organic solutions separately in two zirconia jars of 120 ml. The weight ratio of the TE particles to the organic solution and that of ball to the ink weight were 5 : 1 and 4 : 1, respectively. This ball-to-TE particle ratio was used to ensure uniform milling (where the radius and weight of a ball were 10 mm and 3 g, respectively). Afterward, the jars were sealed and flushed with a steady, gentle stream of nitrogen gas (N_2_) for 5 min. Finally, the final mixtures were ball-milled using a Fritsch Planetary Mill PULVERISETTE 5 premium line at 350 rpm for 60 min. The produced inks were stored under inert conditions for subsequent printing. The dielectric ink was also prepared similarly to the TE ink. The glass dielectric powder was prepared according to the requirement with a 25 wt% solution. Then, these jars were sealed, and the mixture was ball-milled at 350 rpm for 60 minutes to achieve a homogenous dispersion. The ink viscosity is optimized and maintained within the range of 4000–5000 mPa s to ensure printing of a uniform film and compatibility with multi-layer deposition. Finally, the ink was stored in a sealed container.

### 2.3. Fabrication of the 3D print-TEGs

**Fig. 2:**
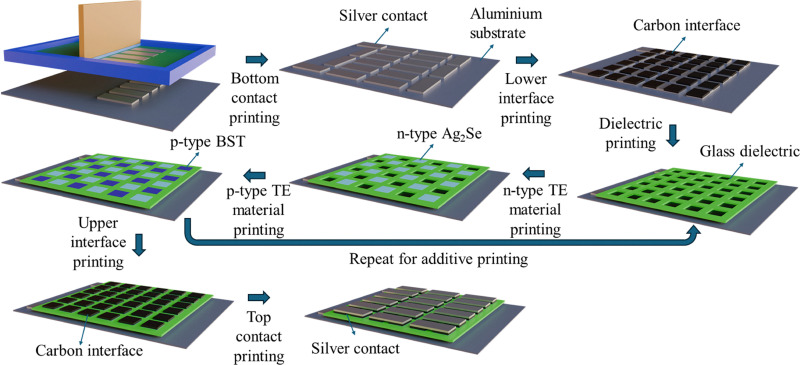
Schematic representation of print-TEG fabrication steps using screen printing.

The n- and p-type printable TE materials were employed to fabricate two 3D print-TEGs with a different number of thermocouples: (a) print-TEG I with 18 thermocouples and (b) print-TEG II with 50 thermocouples (*cf.* Fig. S1(a)–(c), ESI†). The print-TEGs were printed on an anodized aluminium substrate *via* layer-by-layer printing of the bottom silver electrode, carbon interlayer, glass dielectric filler, n- and p-type legs, carbon interlayer, and finally, the top silver electrode. The printing was done using a semi-automated RokuPrint flatbed screen-printing machine equipped with a ‘Sieb 600 × 300 90–40 Y22 Hitex’ polyester mesh screen. This screen configuration, combined with the ink rheology, offers suitable resolution and enables multi-layer alignment with a positional accuracy of ±200 μm. To ensure sufficient interlayer adhesion, each set of 3/4 wet-on-wet printed layers was dried before depositing the subsequent layers. This manufacturing technique offers scalability and allows for design versatility. The schematic of the fabrication process of the print-TEGs is illustrated in Fig. 2: a mesh fabric in a screen is put above the substrate, and a blade presses the ink through the mesh on the substrate, forming a printed layer. Each print-TEG used six different layouts to print five different inks sequentially. Each printed layout was dried on the hot plate, followed by careful alignment and sequential printing of the next layers. A ∼10 μm thick conductive silver bottom electrode was printed on the anodized Al substrate having a 65 μm thickness. A perovskite-grade carbon interface layer of ∼10 μm was then printed on the silver electrode, which reduces the interfacial resistance between the TE legs and the conductive electrode. An empirical investigation was conducted to identify a suitable printable interface material for reducing contact resistance in a printed TEG (*cf.* Table S4, ESI†). The two-probe measurement was taken from the top of the conductive silver to the printed p-type material. The carbon interface enables the lowest internal resistance compared to other interface materials.

In the third step, a glass dielectric ink was printed, which acts as an insulating layer and also gives mechanical strength to the print-TEGs. In the fourth step, n-type and p-type TE legs were printed and dried sequentially, filling up the unfilled space left in the glass dielectric layer. Printing of the dielectric, n-type, and p-type layers was continued until they reached a thickness of >600 μm, forming a 3D structure. Then, a second interlayer was printed on top of the n-type and p-type layers. Lastly, the ∼10 μm thin top silver contact was printed and dried. This screen-printed 3D TEG was then sintered in a vacuum oven at 623 K to ensure that all printed layers were bonded and sintered properly. The final device was then encapsulated with carbon tape, as shown in Fig. 5(c), to protect its structure and improve stability during testing. The thicknesses of print-TEG I and print-TEG II are estimated to be 620 μm and 780 μm, with resistances of ∼3.6 Ω and ∼15 Ω, respectively.

### 2.4. Characterization techniques for the printed materials and print-TEGs

The respective inks were screen-printed in a square shape on a uniform-thickness glass substrate, then sintered at 623 K in an inert atmosphere to study the thermo-physical properties. The temperature-dependent TE parameters, electrical conductivity (*σ*), and Seebeck coefficient (*S*) of the n- and p-type printed materials were studied using a Linseis HCS 10. Temperature-dependent thermal conductivities (*κ*) of 10 mm printed discs made of the p- and n-type inks, followed by sintering at 623 K in N_2_, were determined using a Netzsch LFA-427. The densities of the p-type and n-type printed materials are measured to be 5.1 g cm^−3^ and 5.9 g cm^−3^, respectively. The relative errors associated with the *S*, *σ*, and *κ* measurements were 7%, 6%, and 10%, respectively. The Hall coefficient *R*_H_, Hall mobility *μ*_H_, and charge carrier concentration *n*_H_ of the printed materials were determined by the Van der Pauw method. The microstructural and morphological analyses of the n- and p-type printed legs were done using a JEOL CarryScope JCM-5700 scanning electron microscope (SEM). Microscopic studies were conducted at different magnifications, focusing both on the interior of the legs and the interface between the dielectric and the legs. The objective of this analysis was to assess the print quality and gain an understanding of the TEG structure. We have characterized the TEGs using the maximum power point tracking method by varying the current *I* from −15 mA to +15 mA and temperature differences Δ*T* from 4 K to 43 K. The print-TEGs were clamped between two copper blocks with specific temperatures controlled by two Peltier coolers to maintain a temperature difference Δ*T* across the TEGs. Thermal paste was applied to both sides of the print-TEGs to ensure uniform heat flow through the device, maintaining the predefined Δ*T*. Thermocouples in the copper plates recorded the temperatures at the two surfaces for accurate temperature measurements.

## 3. Experimental results

### 3.1. Microstructural and morphological properties of printed TE-legs

**Fig. 3:**
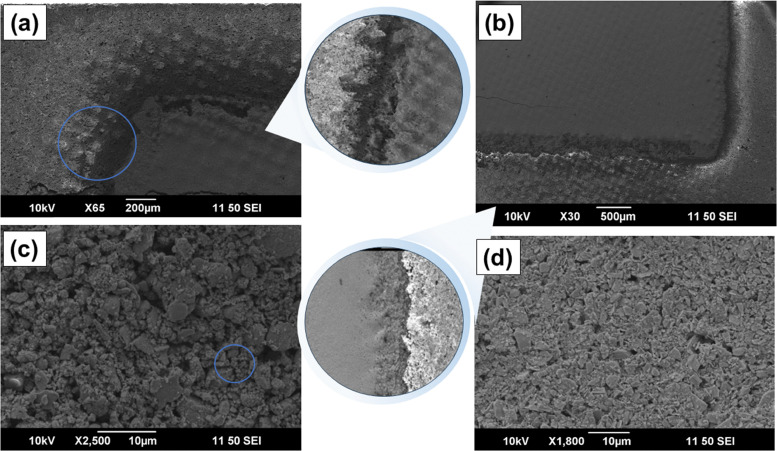
SEM micrographs of (a) printed Ag_2_Se and dielectric filler (brighter), (b) printed BST and dielectric filler (brighter), (c) n-type printed Ag_2_Se leg, and (d) p-type printed BST leg.

The SEM images of the printed n-type Ag_2_Se and p-type BST legs are shown in Fig. 3. The surfaces of both printed legs were found to be smooth at lower magnification. Fig. 3(a) and (b) show the interface between the printed TE legs and the printed dielectric filler material. The corners of the cubic TE legs and their interfaces with the dielectric appear to be sharp without a substantial interdiffusion across the interface. These results demonstrate that the ink was printed homogeneously with a small overlap. Fig. 3(c) and (d) show higher magnification SEM images of the n- and p-type printed legs. This indicates that both printed legs exhibit some porosity. These images also reveal that the particle sizes of n-type Ag_2_Se and p-type BST materials range from 2 to 10 μm. In both images, the samples exhibit good uniformity without cracks, contributing to high performance.

### 3.2. Performance of the n- and p-type printed TE legs

**Fig. 4:**
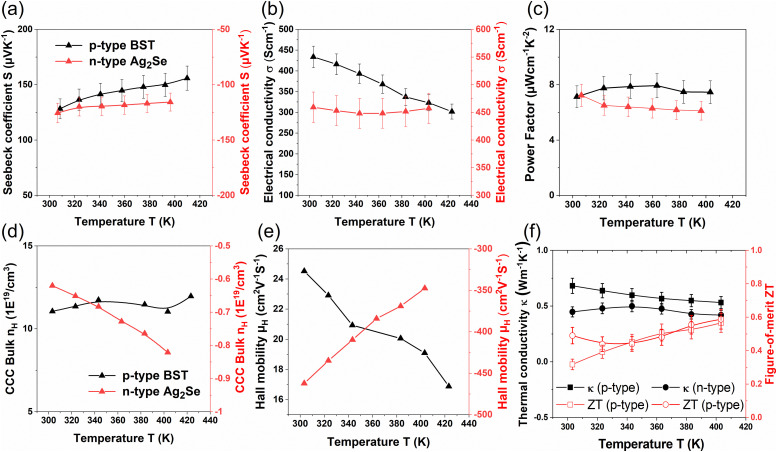
Variation of the TE properties of p- and n-type legs *vs.* temperature. (a) Seebeck coefficient *S*, (b) electrical conductivity *σ*, (c) power factor *S*^2^*σ*, (d) carrier concentration, *n*_H_ (e) Hall mobility *μ*_H_, and (f) thermal conductivity *κ* and figure-of-merit *ZT*.

Temperature-dependent TE performances of the printed n- and p-type legs were studied from 300 K to 400 K (*cf.*Fig. 4). Both the p- and n-type printed materials show |*S*| > 120 μV K^−1^ at room temperature (*cf.*Fig. 4(a)). The *σ* of the printed p-type and n-type legs amounts to 434 S cm^−1^ and 450 S cm^−1^ at room temperature, respectively. The p-type material shows a metallic behavior, while its n-type counterpart exhibits a semiconducting nature at elevated temperatures without a significant change (*cf.*Fig. 4(b)). The *S* for the printed n-type material decreases with increasing temperature due to an increase in the charge carrier concentration. However, due to a decrease in the Hall mobility, *σ* does not change significantly in the complete temperature range (*cf.*Fig. 4(d) and (e)). On the other hand, the *S* is found to increase, while *σ* decreases in the printed p-type material at elevated temperatures. It can be observed that the charge carrier concentration *n*_H_ of the p-type material does not change significantly in the complete temperature range. In contrast, the Hall mobility *μ*_H_ decreases with increasing temperature. Hence, the thermo-transport behavior for both TE properties *S* and *σ* is mainly influenced by the Hall mobility, which depends on charge carrier scattering. The power factor *S*^2^*σ* for the n- and p-type printed material was calculated as a function of temperature. Power factor values of ∼7.4 μW cm^−1^ K^−2^ for n-type and ∼7.13 μW cm^−1^ K^−2^ for p-type printed materials are achieved at room temperature.

Although the power factor values of the printed p-type and n-type materials are relatively good for printed TE materials, they remain lower than those of conventional bulk or hot-pressed counterparts. This difference can be primarily attributed to the lower sintering temperature (623 K) and the pressure-free fabrication process, which results in reduced material density and the presence of organic ingredients. The measured densities of the printed p-type and n-type legs are approximately 5.1 g cm^−3^ and 5.9 g cm^−3^, respectively, compared to their theoretical densities of 6.5 g cm^−3^ and 8.2 g cm^−3^—indicating nearly 22% and 28% porosity in the p-type and n-type printed legs, respectively. This porosity, along with decomposed organic residues, significantly lowers both the electrical and thermal conductivities relative to their bulk values. The pores act as scattering centers, disrupting the transport of both charge carriers and phonons. Hence, the mobility is reduced considerably. The charge carrier concentration remains comparable to that of hot-pressed bulk materials, but the mobility decreases substantially in both n- and p-type printed materials.^30,31^ Furthermore, the scattering of phonons due to micropores and residual organic ingredients reduces the lattice thermal conductivity significantly, resulting in an overall lower thermal conductivity (*κ*). The *κ* is measured to be 0.45 W m^−1^ K^−1^ and 0.68 W m^−1^ K^−1^ at room temperature for n-type and p-type, respectively—values that are more than 50% lower than those of their bulk counterparts (*cf.*Fig. 4(f)). The figure of merit (*ZT*) was calculated using the measured power factors and thermal conductivities. The highest *ZT* values of 0.53 for the p-type material and 0.59 for the n-type material were achieved at 400 K (*cf.*Fig. 4(f)).

### 3.3. Performance of the 3D print-TEGs

**Fig. 5:**
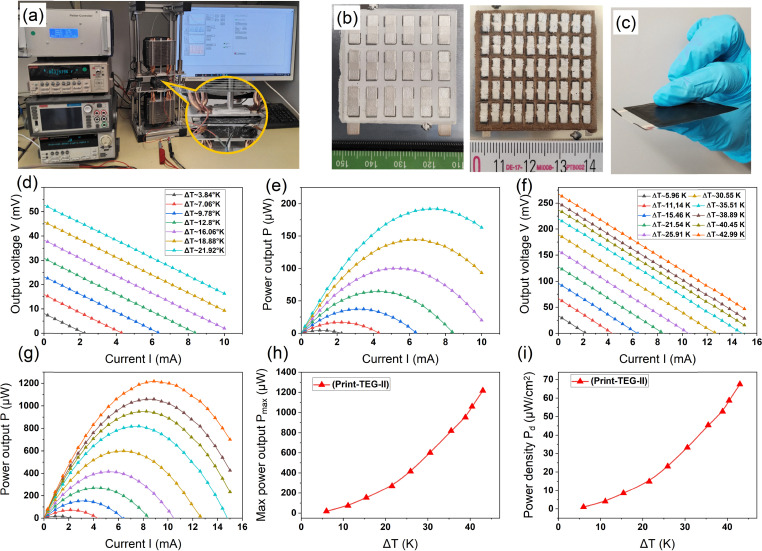
(a) Experimental setup for *V*–*I* characterization using the SMU unit. (b) Top view of the print-TEG I & print-TEG II devices for visual representation. (c) Lateral view of the print-TEG II insulated with carbon tape. (d) *V*–*I* characteristics of print-TEG I at Δ*T*s. (e) *P*–*I* characteristics of print-TEG I at Δ*T*s. (f) *V*–*I* characteristics of print-TEG II at Δ*T*s. (g) *P*–*I* characteristics of print-TEG II at ΔTs. (h) Max power output *P*_max_ of print-TEG II at Δ*T*s. (i) Power density *P*_d_ of print-TEG II at Δ*T*s.

The n- and p-type printed materials were used as prepared to fabricate two different fully printed planar TEGs: (a) print-TEG I and (b) print-TEG II (*cf.*Fig. 5(b) and (c)), with different numbers of thermocouples and thicknesses.

The detailed fabrication processes and characterization techniques have been described in Sections 2.3 and 2.4. The TEG characterization setup, together with a print-TEG connected to an SMU, is shown in Fig. 5(a). When a temperature gradient is applied across two sides, charge carriers in the n-type and p-type materials move from the hot side to the cold side, generating a voltage across the print-TEGs. The output voltages were recorded with the applied current through the devices to study the *V*–*I* characteristics of the print-TEGs. The performances of print-TEG I and print-TEG II were characterized by sweeping the current from −15 mA to 15 mA, as shown in Fig. 5(d)–(g). The maximum Δ*T* applied for print-TEG I is ∼22 K, and that for print-TEG II is ∼43 K. Due to the higher thickness of print-TEG II, it could maintain a higher Δ*T*. The power output *P* and open circuit voltage *V*_OC_ for both print-TEGs increases with increasing Δ*T*. Print-TEG I reaches an open circuit voltage of ∼52.8 mV, exhibiting a maximum power output of 192 μW at a Δ*T* of ∼22 K (*cf.*Fig. 5(d) and (e)). The print-TEG II, in contrast, exhibits a higher open circuit voltage of ∼128 mV at the same Δ*T* of ∼22 K (*cf.*Fig. 5(f) and (g)). Moreover, for a temperature difference Δ*T* ∼ 43 K, the print-TEG II shows a *V*_OC_ of ∼268 mV and an impressive output power *P*_max_ of ∼1.22 mW. This corresponds to a maximum power density *P*_d_ of 67 μW cm^−2^ (*cf.*Fig. 5(h) and (i)). To the best of our knowledge, this is the highest reported power output for fully printed planar TEGs. The detailed power density comparison between print-TEG I and print-TEG II as a function of Δ*T* is shown in Fig. S3 (ESI†). The *V*_OC_ and *P*_max_ for both print-TEGs increase with increasing Δ*T* (*cf.* Fig. S2(d) and S3(a), ESI†). As the number of thermocouples for print-TEG II is higher, it shows a higher output voltage for all Δ*T* according to the following equation;*V*_OC_ = *N*·(*S*_p_ − *S*_n_)·Δ*T*where ‘*N*’ is the number of thermocouples and *S*_p_ − *S*_n_ is the total Seebeck coefficient per thermocouple of the print-TEGs. The number of thermocouples in print-TEG II is 2.78 times higher than in print-TEG I. The open circuit voltage of ∼128 mV is slightly lower than the theoretical prediction, but it is approximately ∼2.42 times higher in print-TEG II than print-TEG I at Δ*T* ∼ 22 K. This is attributed to the higher thermal resistance in print-TEG II, which reduces the Seebeck coefficient per thermocouple (*cf.* Fig. S2, ESI†). The internal resistance of print-TEG II, however, increases to 15 Ω as compared to 3.6 Ω for print-TEG I. Therefore, although the *V*_OC_ increases with the number of thermocouples, the overall maximum power output *P*_max_ is not altered significantly. The print-TEG I and TEG II exhibit a maximum power density *P*_d_ of 12 μW cm^−2^ and 14.9 μW cm^−2^, respectively, at Δ*T* ∼ 22 K. It can be observed that thickness plays an important role in maintaining a higher temperature Δ*T*, and thus achieving higher power output. Further increases in print-TEG thickness can be realized by using screens with lower mesh density and higher thread diameter, along with adjustments to ink rheology. However, greater thickness may also increase the brittleness of the print-TEG. This drawback can be mitigated by employing a high-temperature dielectric material with improved adhesion and mechanical flexibility, such as a polyimide-based dielectric.

**Fig. 6:**
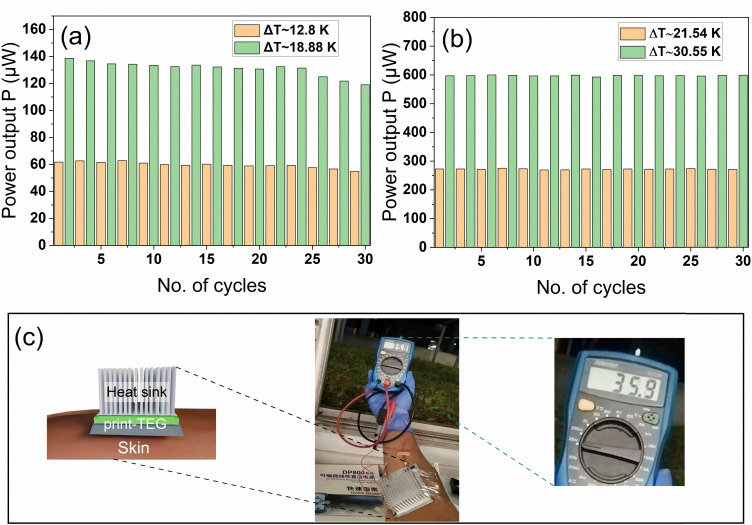
Comparison of print-TEG I and print-TEG II. (a) Power output of print-TEG I at different cycles. (b) Power output of print-TEG II at different cycles. (c) Demonstration of the print-TEG II, integrating it onto an arm.

For long term energy harvesting applications, it is paramount to test the stability of the print-TEGs for extended cycling. A stability test has been conducted for both print-TEGs by running them for 30 consecutive cycles at two different Δ*T*s as shown in Fig. 6(a) and (b). Each cycle, as shown in the figure, is the result of alternating 5 current sweeps. We observe a stable output power of about ∼60 μW at Δ*T* ∼ 13 K and ∼140 μW at Δ*T* ∼ 19 K for print-TEG I and ∼270 μW at Δ*T* ∼ 21.5 K and ∼600 μW at Δ*T* ∼ 30.5 K for print-TEG II. The resulting power output of print-TEGs with standard deviations of ∼7.6 × 10^−6^, 1.7 × 10^−5^ and 1.49 × 10^−6^, 1.7 × 10^−6^ indicates a high stability. A demonstration to test the applicability of print-TEGs was carried out by integrating the print-TEG II onto a human arm (*cf.*Fig. 6(c)). A heat sink was attached to the top of the print-TEG II to ensure good convective cooling. An infrared thermometer (TFA ScanTemp 410) is used to determine the body temperature and the temperature of the heat sink. An output voltage of ∼36 mV is generated by the print-TEG II for a temperature difference of 5 K.

## 4. Discussion

### 4.1. The simulation results of the print-TEGs

The print-TEG II was selected for simulation to compare its performance with experimental results. The model was set up using PTC's Creo Parametric 10.0 CAD software and subsequently imported into COMSOL Multiphysics 6.2 for simulation. COMSOL Multiphysics was selected for this analysis due to its capability to handle multiple coupled physics phenomena. Within the simulation environment, appropriate material properties were assigned to all materials of the 3D print-TEG II: anodized aluminum as the substrate, n-type Ag_2_Se, and p-type Bi_0.5_Sb_1.5_Te_3_ as the TE materials, carbon paste as a diffusion barrier between the electrodes and TE materials, silver as the electrode, glass as both a dielectric and filler material for structural support, and carbon tape to encapsulate the top portion of the device. Structural parameters of the 3D print-TEG II device used in COMSOL are given in Table S1 (ESI†). The physics, including heat transfer in solids and electric current densities, and the electrical circuit, was defined for the different elements of the TEG.

**Fig. 7:**
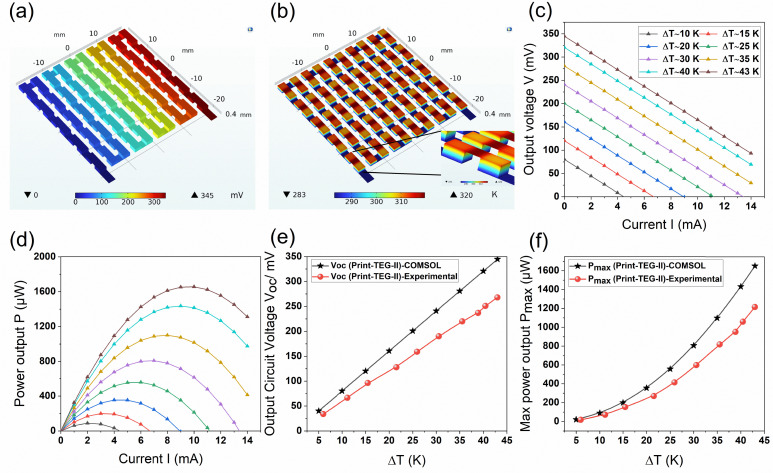
COMSOL simulated model of print-TEG II. (a) Electric potential map of print-TEG II. (b) Thermal map of print-TEG II. (c) Simulated *V*–*I* characteristics at different temperatures. (d) Simulated *P*–*I* characteristics at different temperatures. (e) Comparison of open circuit voltage, simulated *vs.* experimental. (f) Comparison of Max power output, simulated *vs.* experimental.

We employed a structured mesh with extremely fine element size to ensure the accuracy of the simulations. A fully coupled solver was used in these simulations to address this Multiphysics problem effectively. Fig. 7 illustrates the simulation results, including 3D temperature and electric potential plots, *V*–*I*, *P*–*I*, *V*–Δ*T*, and *P*–Δ*T* curves, respectively. In COMSOL, six temperature differences were applied to see the voltage distribution inside the print-TEG II. The resulting voltage and power output from it are shown in Fig. 7(c) and (d). From the simulated *V*–*I* characteristics an open circuit voltage *V*_OC_ of 344 mV is observed at Δ*T* = 43 K. This agrees well with the experimentally observed value of 268 mV. Moreover, the experimental temperature difference dependence agrees with our simulations. The minor differences between simulation and experimental results can be explained by a higher thermal resistance due to imperfect mechanical contact and the presence of an air gap between the TEG and measurement setup than what was considered in the COMSOL simulation. Therefore, the experimental *V*_OC_ is lower than the simulated value, reducing power output (*cf.*Fig. 7(f)). The real device also possesses some imperfections due to printing roughness, porosity, and fabrication errors, which are not accounted for in the model. The printed-TEGs presented in this investigation utilize identical cross-sectional areas for both n-type and p-type TE legs. However, due to the mismatch in their TE properties, the optimal leg areas for maximum power output can be adjusted accordingly.^32,33^ To investigate this aspect further, a thermocouple within the device is geometrically optimized to achieve maximum power density. The resulting power density contours are shown in Fig. S4 (ESI†), plotted as a function of TE leg thickness and the geometric factor (GF = cross-sectional area of the p-type leg (*A*_p_)/cross-sectional area of the p-type material (*A*_n_)). The increase in performance with TE thickness for printed TEGs is attributed to lower parasitic heat flow through the filler material as well as maintained Δ*T* across its side. It is also evident that the power density reaches its maximum value when the GF lies between 0.2–0.3, indicating that the *A*_p_ should be approximately three to five times smaller than *A*_n_. Therefore, at a constant n-type material area, the fill factor of the device is slightly reduced due to the decrease in the p-type leg area. At this geometric optimization condition, the power density could improve by approximately ∼39%, increasing from 106 μW cm^−2^ to 147 μW cm^−2^.

### 4.2. Reproducibility of the print-TEGs

**Fig. 8:**
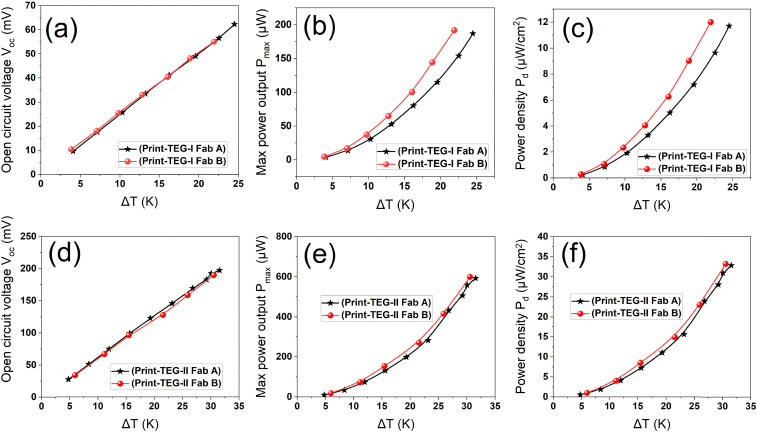
Performance of print-TEGs Fab A & Fab B. (a) *V*_OC_ of print-TEG I at Δ*T*s. (b) *P*_max_ of print-TEG I at Δ*T*s. (c) *P*_d_ of print-TEG *I* at Δ*T*s. (d) *V*_OC_ of print-TEG II at Δ*T*s. (e) *P*_max_ of print-TEG II at Δ*T*s. (f) *P*_d_ of print-TEG II at Δ*T*s.

To scale up print-TEG production, ensuring reproducible performance is crucial. To check the consistency of the fabrication process of the print-TEGs, two additional sets (Fab A and Fab B) of the print-TEG I and print-TEG II were fabricated and characterized (*cf.*Fig. 8). The resistances of the two print-TEG I devices, with thicknesses of 600 μm and 620 μm, are found to be 4.97 Ω and 3.6 Ω, respectively. Similarly, the resistances of the two print-TEG II devices, with thicknesses of 720 μm and 780 μm, are found to be 19 Ω and 15 Ω, respectively. The open circuit voltages of both print-TEG sets (Fab A & Fab B) are plotted against different Δ*T*s in Fig. 8(a) and (d), showing similar *V*_OC_s. On the other hand, the power output of the print-TEG I in Fab B is slightly higher than that of Fab A because of lower electrical resistance values.

Whereas the print-TEG II exhibits nearly identical power outputs for both Fab A and Fab B. These results demonstrate the high reproducibility of the print-TEG fabrication process. The performance of all the print-TEGs is summarized in Fig. S3 and Table S2 (ESI†).

### 4.3. Normalized power density output and manufacturing cost analysis

**Fig. 9:**
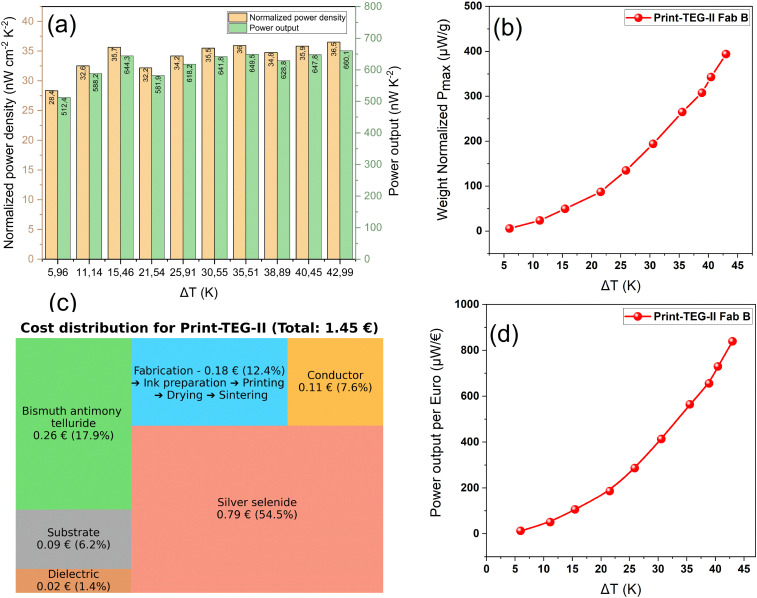
(a) Normalized power density and power output/Δ*T*^2^. (b) Cost distribution for print-TEG II. (c) Fabrication cost distribution for print-TEG II. (d) Power output per Euro (μW per €) at Δ*T*s.

We have also calculated the area- and mass-normalized power density of the print-TEGs (*cf.* Fig. S3, ESI†). The print-TEG II exhibits a normalized power density of 36 nW cm^−2^ K^−2^ for Δ*T* = 43 K, which is the highest reported value for a fully printed planar TEG (*cf.*Fig. 9(a)). A detailed comparison of the print-TEG II performance with the previously reported fully printed TEGs is presented in Fig. 1(a). While some in-plane printed TEGs exhibit high power densities, they generate less than 100 μW, which is insufficient for practical applications. In contrast, the print-TEG II achieves 1.22 mW under similar conditions—a power output not previously reported. In addition, the print-TEG II demonstrates an impressive power output per unit mass, generating 400 μW g^−1^ (*cf.*Fig. 9(b)). This signifies the potential of print-TEGs as lightweight power sources, and just 1 gm of print-TEG II could be sufficient to power low-power IoT devices. Finally, we present the total fabrication cost and percentage distribution of each component and step involved (*cf.*Fig. 9(c)). In this analysis, the cost of the substrate, bulk TE material (Ag_2_Se and BST), interface carbon paste, conductive silver paste, glass dielectric, and fabrication presented in Table S3 (ESI†) were considered. We also plotted power obtained from the print-TEG II per Euro (μW per €) at different Δ*T*s in Fig. 9(d). After geometric optimization of the print-TEG II by reducing p-type-while maintaining the same amount of n-type material, its total cost decreases due to less material being used. Fig. S4(c) and (d) (ESI†) shows the cost difference between the current scenario and geometrically optimized print-TEG II. An approximate 18% cost reduction, from 1.45 € per TEG to 1.19 € per TEG, can be achieved. A more detailed understanding of cost optimization can be drawn from recent works that provide detailed methodologies for evaluating material efficiency, process scalability, and cost-per-watt metrics for additive manufacturing approaches.^32,34^

It should be noted that the cost analysis pertains to the fabrication of a laboratory-scale prototype. Although the layer-by-layer additive screen-printing of a single 3D TEG may appear complex, it highlights strong potential for scalable manufacturing. In particular, this fabrication method is compatible for roll-to-roll manufacturing, facilitating high-throughput, continuous production of a large number of printed TEGs simultaneously. Therefore, additive screen-printed 3D TEGs offer distinct advantages over conventional 3D printed and bulk TEGs, including scalability, cost-effectiveness, and shape conformability.

## 5. Conclusions

In conclusion, the long-standing promise of low-cost TEGs as battery replacements for low-power electronics is challenged by the fact that batteries can sustain devices consuming less than 100 μW for several months. However, for devices operating in the hundreds of microwatts range, batteries deplete quickly, requiring frequent replacements. This study presents fully printed 3D TEG modules with the potential to serve as alternatives to Li-ion batteries for wearables and IoT devices. Using an additive screen-printing process, we fabricated two 3D planar print-TEGs with varying thermocouple counts, addressing key limitations such as high contact resistance and low thickness associated with screen printing. The print-TEG with 50 thermocouples achieved a maximum power output of 1.22 mW and an open-circuit voltage of 268 mV at Δ*T* = 43 K, with a peak power density of 67 μW cm^−2^, setting a benchmark for fully printed planar TEGs. These results highlight the potential of print-TEGs as a reliable and maintenance-free power source for wearables and IoT applications.

## Conflicts of interest

There are no conflicts to declare.

## Supplementary Material

EE-018-D5EE01151E-s001

## Data Availability

The data that support the findings of this study are provided in the article and its supporting information document. The data are also available from the corresponding authors upon request.

## References

[cit1] Garg N., Garg R. (2018). Proc. Int. Conf. Intell. Sustainable Syst. ICISS, 2017.

[cit2] Wei Z., Zhao J., He H., Ding G., Cui H., Liu L. (2021). J. Power Sources.

[cit3] Wu K. K., Wang H. Y., Chen C., Tao T. (2022). Microelectron. J..

[cit4] Xiao J., Niu B., Lu J., Hong J., Zhou T., Xu Z. (2024). Chem. Eng. J..

[cit5] Costa C. M., Barbosa J. C., Gonçalves R., Castro H., Campo F. J. D., Lanceros-Méndez S. (2021). Energy Storage Mater..

[cit6] Vinet L., Zhedanov A. (2011). J. Phys. A: Math. Theor..

[cit7] Kramer L. R., Maran A. L. O., De Souza S. S., Ando O. H. (2019). Energies.

[cit8] Zhu Y., Xu W., Ravichandran D., Jambhulkar S., Song K. (2021). J. Mater. Chem. A.

[cit9] Hasan M. N., Nafea M., Nayan N., Mohamed Ali M. S. (2022). Adv. Mater. Technol..

[cit10] Rösch A. G., Gall A., Aslan S., Hecht M., Franke L., Mallick M. M., Penth L., Bahro D., Friderich D., Lemmer U. (2021). npj Flexible Electron..

[cit11] Shakeel M., Rehman K., Ahmad S., Amin M., Iqbal N., Khan A. (2021). Renewable Energy.

[cit12] Burton M., Howells G., Atoyo J., Carnie M. (2022). Adv. Mater..

[cit13] Mallick M. M., Rösch A. G., Franke L., Ahmed S., Gall A., Geßwein H., Aghassi J., Lemmer U. (2020). ACS Appl. Mater. Interfaces.

[cit14] Franke L., Georg Rösch A., Khan M. I., Zhang Q., Long Z., Brunetti I., Joglar M. N., Lara A. M., Simão C. D., Geßwein H., Nefedov A., Eggeler Y. M., Lemmer U., Mallick M. M. (2024). Adv. Funct. Mater..

[cit15] Rösch A. G., Franke L., Mallick M. M., Lemmer U. (2023). Energy Convers. Manage..

[cit16] Madan D., Wang Z., Wright P. K., Evans J. W. (2015). Appl. Energy.

[cit17] Iezzi B., Ankireddy K., Twiddy J., Losego M. D., Jur J. S. (2017). Appl. Energy.

[cit18] Mallick M. M., Franke L., Rösch A. G., Geßwein H., Long Z., Eggeler Y. M., Lemmer U. (2022). Adv. Sci..

[cit19] Brunetti I., James Pataki N., Hinojosa D. R., Hawkey A., Karakaya O., Rainer C., Khan M. I., Franke L., Mallick M. M., Hernandez-Sosa G., Kemerink M., Caironi M., Lemmer U. (2024). Adv. Mater. Technol..

[cit20] Mallick M. M., Franke L., Hussein M., Rösch A. G., Long Z., Eggeler Y. M., Lemmer U. (2024). Small Sci..

[cit21] Cui G. P., Feng C. P., Xu S. C., Sun K. Y., Ji J. C., Hou L., Lan H. B., Shang H. J., Ding F. Z. (2024). ACS Appl. Mater. Interfaces.

[cit22] Hwang S., Jang D., Lee B., Ryu Y. S., Kwak J., Kim H., Chung S. (2023). Adv. Energy Mater..

[cit23] Zhang X., Hou Y., Yang Y., Wang Z., Liang X., He Q., Xu Y., Sun X., Ma H., Liang J., Liu Y., Wu W., Yu H., Guo H., Xiong R. (2023). Adv. Mater..

[cit24] Kim S. J., Lee H. E., Choi H., Kim Y., We J. H., Shin J. S., Lee K. J., Cho B. J. (2016). ACS Nano.

[cit25] Sarbajna A., Rösch A. G., Franke L., Lemmer U., Mallick M. M. (2023). Adv. Eng. Mater..

[cit26] Burton M. R., Howells G., Mehraban S., Mcgettrick J. D., Lavery N., Carnie M. J. (2023). ACS Appl. Energy Mater..

[cit27] Kato K., Kuriyama K., Yabuki T., Miyazaki K. (2018). J. Phys.:Conf. Ser..

[cit28] Mytafides C. K., Tzounis L., Karalis G., Formanek P., Paipetis A. S. (2021). J. Power Sources.

[cit29] Brunetti I., Ferrari F., Pataki N. J., Abdolhosseinzadeh S., Heier J., Koster L. J. A., Lemmer U., Kemerink M., Caironi M. (2024). Adv. Mater. Technol..

[cit30] Liu Y., Lu Y., Wang Z., Li J., Wei P., Zhao W., Chen L., Cai K. (2022). J. Mater. Chem. A.

[cit31] Wei Z., Wang C., Zhang J., Yang J., Li Z., Zhang Q., Luo P., Zhang W., Liu E., Luo J. (2020). ACS Appl. Mater. Interfaces.

[cit32] Zhou Y., Liu X., Jia B., Ding T., Mao D., Wang T., Ho G. W., He J. (2023). Sci. Adv..

[cit33] Mao D., Zhou Y., Yu Y., Wang Y., Han M., Meng Q., Lu Y., Feng J., Kong M., Yang H., Gan Q., Xu X., Xie L., Ho G. W., He J. (2024). Joule.

[cit34] Zhou Y., Ding T., Xu G., Yang S., Qiu C. W., He J., Ho G. W. (2024). Nat. Rev. Phys..

