# Supplementary material for: Milliwatt-scale 3D thermoelectric generators via additive screen printing
Source: Energy Environ Sci. 2025 Jul 4;18(15):7648–59. doi: 10.1039/d5ee01151e (PMC12232290; doi:10.1039/d5ee01151e)
Supplement: EE-018-D5EE01151E-s001 [file EE-018-D5EE01151E-s001.pdf]

Supplementary Information (SI) for Energy & Environmental Science.
This journal is © The Royal Society of Chemistry 2025

Supporting information

Milliwatt-Scale 3D Thermoelectric Generators via Additive Screen Printing

Sairam Antharam[1], Muhammad Irfan Khan[2], Leonard Franke[2], Zirui Wang[2], Nan Luo[2], Jan Feßler[2], Wenjie Xie[1], Uli Lemmer[2,3,] and Md Mofasser Mallick[2,]**

[1]Institut für Materialwissenschaft, Technical University of Darmstadt, 64287 Darmstadt, Germany.

[2]Light Technology Institute, Karlsruhe Institute of Technology (KIT), 76131 Karlsruhe, Germany.

[3]Institute of Microstructure Technology, Karlsruhe Institute of Technology (KIT), 76344 Eggenstein-Leopoldshafen, Germany

*Corresponding authors

Email: uli.lemmer@kit.edu; mofasser.mallick@kit.edu

S1. Structures of print-TEGs

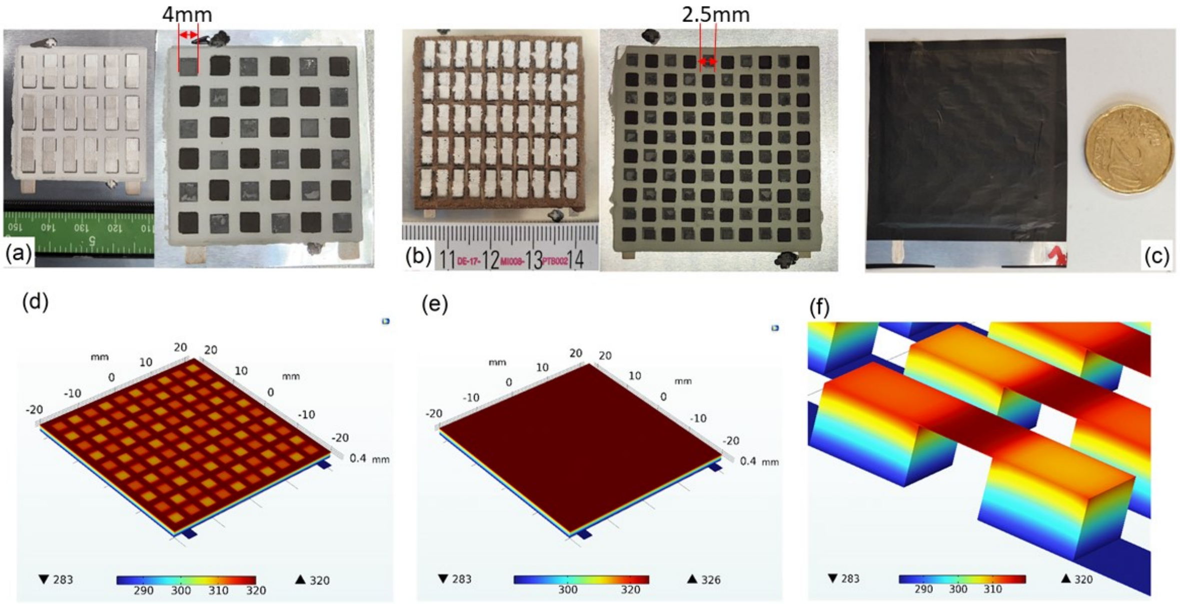

Figure S1: *a) Schematic representation of print-TEG-I with and without top contact b) Schematic representation of print-TEG-II with and without Top contact c) Carbon tape encapsuted print-TEG for characterization (d-f) Schematic representation of thermal map.*

The print-TEG I and print-TEG II with/without top electrodes are shown in Figure S1 (a-b). It can be observed that the print TEG-I and print TEG-II comprise 18 and 50 thermocouples respectively. The dimensions of the TE legs are to be 4 mm x 4 mm and 2.5 mm x 2.5 mm for Print-TEG-I and Print-TEG-II respectively. As we discussed in Section 2.3, Fig S1(c) shows the encapsulated print-TEG. We have encapsulated print-TEG using a 5 micron carbon to avoid electrical shortcut during measurement and achieve better mechanical stability. We simulated the thermal map of print-TEG-II at legs and dielectric in order to analyse heat flow through the different materials. It can be observed that the thermal gradient across the n- and p-type low temperature gradient TE legs is lower compared to that for dielectric filler. This attributes most of the heat flow through the TE legs due to higher thermal conductivity, converting the heat into electricity.

S2. Comparison of thermovoltages of the print-TEGs

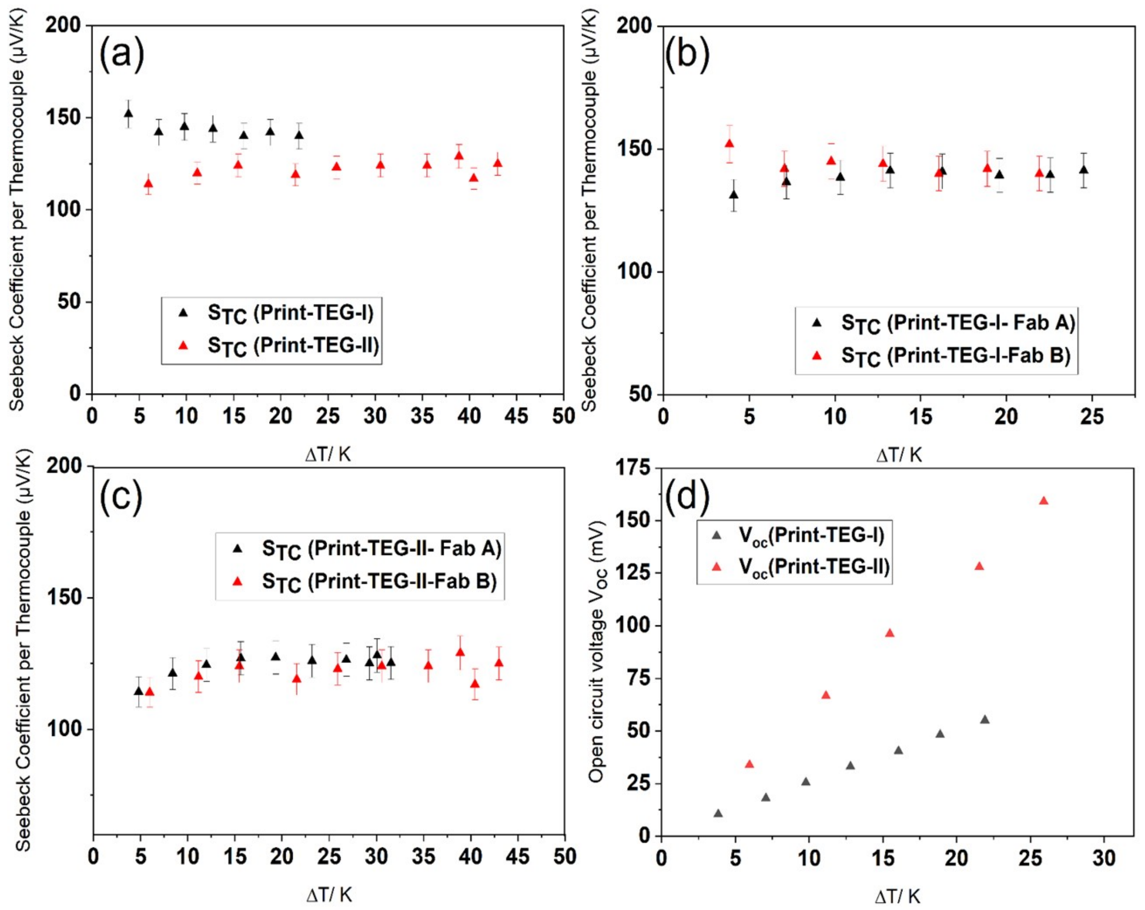

Figure S2: *(a) Seebeck coefficient per thermocouple for print TEG-I and Print TEG-II. (b) Seebeck coefficient per thermocouple for print TEG-I Fab A(600µm)and Fab B(620µm). (c) Seebeck coefficient per thermocouple for print TEG-II Fab A(720µm) and Fab B(780µm). (d) Open circuit voltages for the print-TEG I and print-TEG II.*

As the number of thermocouples in print-TEG-I and print-TEG-II are 18 and 50, respectively, print-TEG-II contains ~2.77 times higher number of thermocouples than print-TEG I. Theoretically, this increase in the number of thermocouples should lead to similar increase in the open-circuit voltage. However, from Fig. S2(a), we observe that the effective Seebeck coefficient of print-TEG-II is lower than that of Print-TEG-I. This explains the reduction in the measured open-circuit voltage of print-TEG-II (128 mV), whereas the expected value based on the equation is 153.05 mV.

$$V_{OC} = N \cdot (S_p - S_n) \cdot \Delta T$$

The reduction thermovoltage in the print-TEGs is probably due to higher thermal resistance. Additionally, Figure. S2 (b) and (c) show that the Seebeck coefficients of both Fab-A and Fab-B both Print-TEG's exhibit similar trends, indicating reproducibility in device properties. This consistency highlights the effectiveness of the printing process in achieving desirable thermoelectric characteristics.

S3. Performance of the print-TEGs

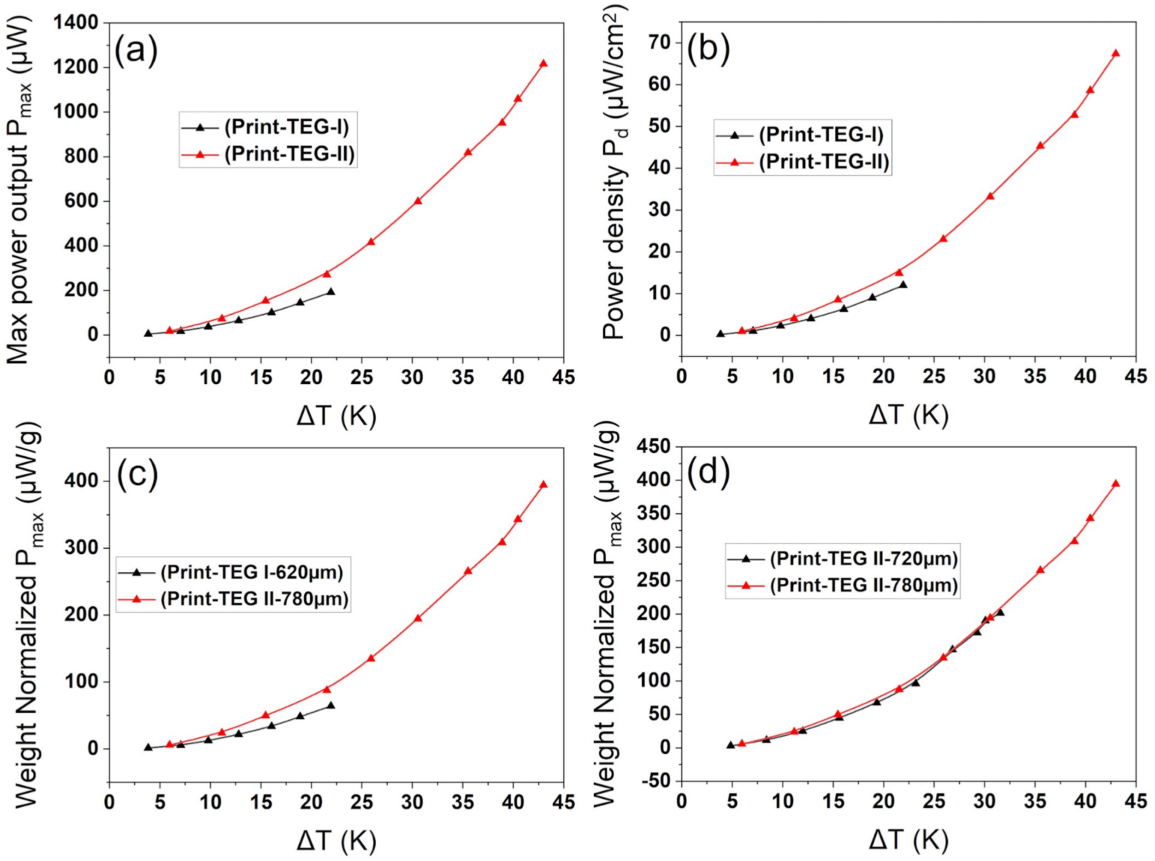

Figure S3: *(a)Max power vs Δ T for print-TEG I and II, (b) Power density vs delta T for print-TEG I and II, (c) Max power per gram for print-TEG I and II, (d) Max power per gram for print-TEG II Fab A and Fab B.*

The voltage characteristics of print-TEG-I and print-TEG-II exhibit a similar trend, with the open-circuit voltage increasing as the number of thermocouples increases. However, due to differences in resistance—3.6 Ω for print-TEG-I and 15 Ω for print-TEG-II—the power output in Figure S3(a) shows only a minimal difference, with print-TEG-II generating 269.8 μW compared to 192 μW for print-TEG-I at ~22K . This effect is also reflected in power density in Figure S3(b) and weight-normalized power output in Figure S3(c). Weight Normalized power output for 780 μm and 720 μm device is observed to be similar, shown in Figure S3 (d).

S4: Geometric optimization and cost analysis of Print-TEG-II

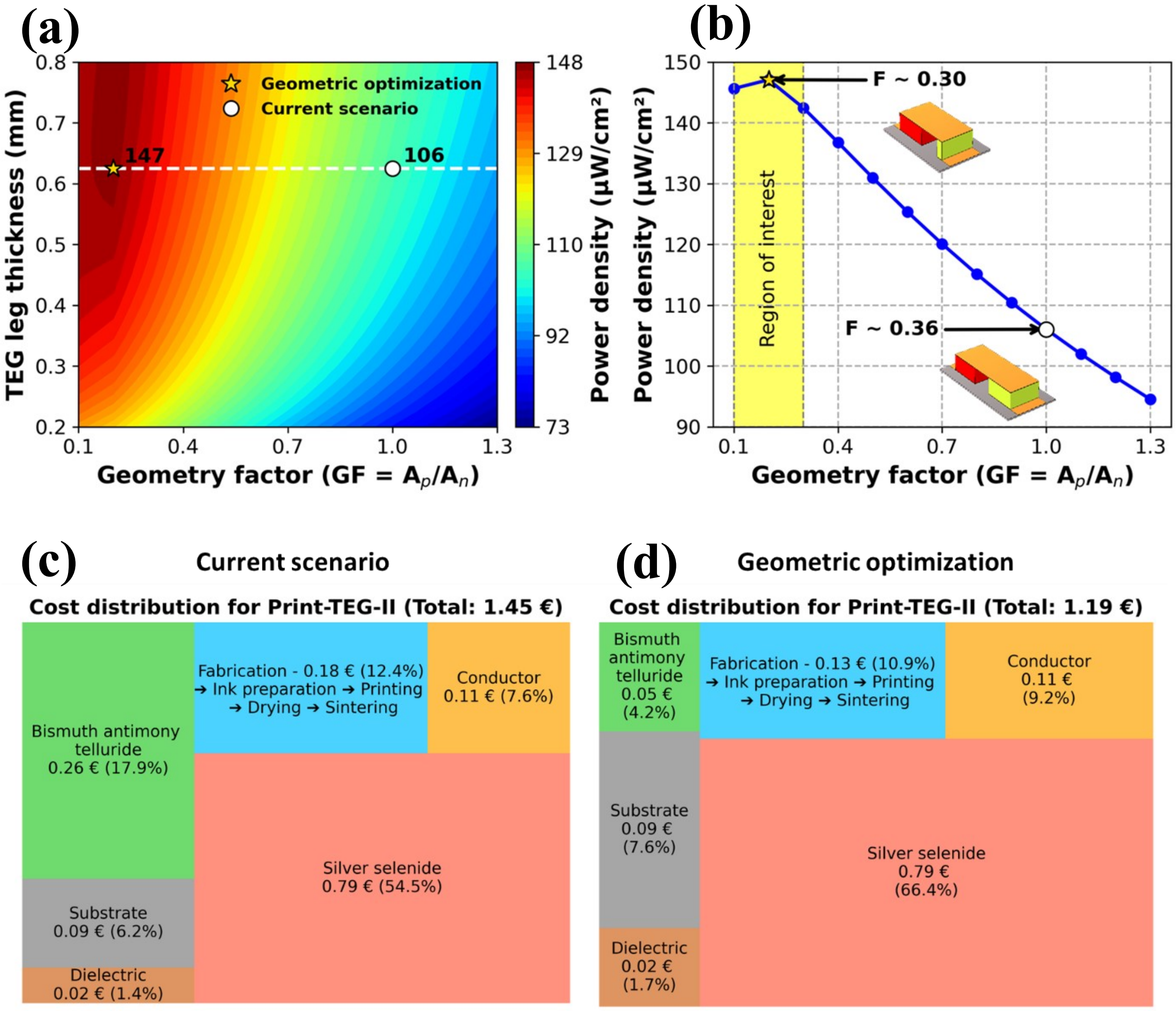

Figure S4: *Geometric optimization and cost analysis of Print-TEG-II, a) Power density contours as function of leg thickness and geometry factor, b) Power density vs geometry factor, c) Cost distribution of current scenario (reported Print-TEG-II), d) Cost distribution of geometrically optimized Print-TEG-II.*

Table S1. Structural parameters of the 3D print-TEG II device used in COMSOL

**Table 1 | Structural parameters of the COMSOL 3D print-TEG-II device.**		
**No. of Layers**	**Material**	**Thickness (μm)**
Layer 1	Anodized aluminum (substrate)	65
Layer 2	Bottom silver conductor	10
Layer 3	Bottom carbon paste	10
Layer 4	TE material (n- and p-type) & Glass dielectric	625
Layer 5	Top carbon paste	10
Layer 6	Top silver conductor	10
Layer 7	Carbon encapsulation	50
**3D print-TEG-II**	**-**	**780**

Table S2:Summarized performance data of print-TEG-I and print-TEG-II

**Print-TEGs**	V_{oc} *in mV*		P_{max} *in* μW		P_d *in* μWcm^{-2}	
	Fab A	***Fab B***	***Fab A***	***Fab B***	***Fab A***	***Fab B***

Print-TEG-I	62.3 (ΔT=22 K)	52.8	187	190	11.7	12
Print-TEG-II	197.5 (ΔT=32 K)	268 (ΔT=43 K)	592 (ΔT=32 K)	1216.5 (ΔT=43 K)	32.8 (ΔT=32 K)	67.4 (ΔT=43 K)

We have summarized the open circuit voltage (V_{oc}), maximum power output (P_{max}) and power density of both devices print TEG-I (Fab-A with 600µm and Fab-B with 620µm) and print TEG-II (Fab-A with 720µm and Fab-B with 780µm). The highest power density of ~67.4µW/cm^2 is observed in Print-TEG-II for ΔT=43 K.

Table S3. TEG manufacturing cost analysis

Table S3: Material and processing costs

**| TEG fabrication cost analysis**		
**Components and/or steps involved**	**Cost**	**Source**
p-type TE material ($Bi_{0.5}Sb_{1.5}Te_3$)	300 €/kg	www.everredtronics.com
n-type TE material (Ag_2Se)	866 €/kg	www.sigmaaldrich.com
Anodized aluminum substrate (Al-Al_2O_3)	125 €/kg	www.steinertglobal.com
Dielectric (Thermally cured insulator)	10 €/kg	www.aksharchemindia.com
Contact material (Ag)	2000 €/kg	www.novacentrix.com
Diffusion barrier (C)	463 €/kg	www.dycotecmaterials.com
Ink preparation (Ball milling)	35.428 €/kg	Own assessment
Printing (Screen printing)	4.629 €/m^2	Own assessment
Drying (Hot plate drying)	40.26 €/kg	Own assessment
Sintering	2.03 €/ m^2	Own assessment

Table S4. TEG interface material

Table S4*: Empirical investigation of a suitable printable interface material to lower contact resistances in a printed TEG device. The two-probe measurement was taken from the top of the conductive silver to the printed p-type material.*

Contact types	Two probe resistivity measurement (Ω), Fluke 289
Ag_2Se/p-type TE leg	15.34
Silver/p-type TE leg	11.54
Silver/PEDOT/ p-type TE leg	1.5
Silver/graphene/ p-type TE leg	0.72
Silver/carbon/ p-type TE leg	0.42

Two primary factors contribute to the high internal resistance of the TEG: (a) low TE material density and (b) high electrode–leg interface resistance. The low material density results from the pressure-free fabrication process, which reduces overall electrical conductivity and increases internal resistance. Applying pressure after printing may help reduce this resistance. Silver ink is commonly used as an electrode material in printed TEGs due to its high conductivity and low curing temperature. However, high contact resistance between printed silver electrodes and Bi-Te-based TE legs remains a significant challenge. This is likely due to surface roughness, porosity, and diffusion of silver into the TE material. In bulk TEGs, metals like Ni and Fe are often used as diffusion barrier layers, but printable inks based on these metals are not readily available. To address this, we investigated the variation in contact resistance using different printable interface materials (see Table S4). Among the tested materials, the carbon interface showed the lowest contact resistance. Notably, omitting the carbon interface increased the resistance by more than 20 times. Further improvements may be possible by replacing carbon ink with more conductive carbon-based printable compounds.
